# Public health preparedness and response synergies between institutional authorities and the community: a qualitative case study of emerging tick-borne diseases in Spain and the Netherlands

**DOI:** 10.1186/s12889-021-11925-z

**Published:** 2021-10-18

**Authors:** Daniel H. de Vries, John Kinsman, Anne Lia Cremers, John Angrén, Massimo Ciotti, Svetla Tsolova, Emma Wiltshire, Judit Takacs

**Affiliations:** 1 University of Amsterdam, Amsterdam, the Netherlands; 2 European Centre for Disease prevention and Control (ECDC), Solna, Sweden; 3 VU University, Amsterdam, the Netherlands; 4 The Swedish Civil Contingencies Agency, Karlstad, Sweden; 5 Centre for Social Sciences, Hungarian Academy of Sciences Centre of Excellence, Budapest, Hungary

**Keywords:** Public health preparedness, Community engagement, Synergies, Lyme Borreliosis, Tick-borne encephalitis, Crimean-Congo Haemorrhagic Fever, European Union

## Abstract

**Background:**

Communities affected by infectious disease outbreaks are increasingly recognised as partners with a significant role to play during public health emergencies. This paper reports on a qualitative case study of the interactions between affected communities and public health institutions prior to, during, and after two emerging tick-borne disease events in 2016: Crimean-Congo Haemorrhagic Fever in Spain, and Tick-Borne Encephalitis in the Netherlands. The aim of the paper is to identify pre-existing and emergent synergies between communities and authorities, and to highlight areas where synergies could be facilitated and enhanced in future outbreaks.

**Methods:**

Documentary material provided background for a set of semi-structured interviews with experts working in both health and relevant non-health official institutions (13 and 21 individuals respectively in Spain and the Netherlands), and focus group discussions with representatives of affected communities (15 and 10 individuals respectively). Data from all sources were combined and analysed thematically, initially independently for each country and then for both countries together.

**Results:**

Strong synergies were identified in tick surveillance activities in both countries, and the value of pre-existing networks of interest groups for preparedness and response activities was recognised. However, authorities also noted that there were hard-to-reach and potentially vulnerable groups, such as hikers, foreign tourists, and volunteers working in green areas. While the general population received preventive information about the two events, risk communication or other community engagement efforts were not seen as necessary specifically for these sub-groups. Post-event evaluations of community engagement activities during the two events were limited, so lessons learned were not well documented.

**Conclusions:**

A set of good practices emerged from this study, that could be applied in these and other settings. They included the potential value of conducting stakeholder analyses of community actors with a stake in tick-borne or other zoonotic diseases; of utilising pre-existing stakeholder networks for information dissemination; and of monitoring community perceptions of any public health incident, including through social media. Efforts in the two countries to build on the community engagement activities that are already in place could contribute to better preparedness planning and more efficient and timely responses in future outbreaks.

## Background

Public health emergency preparedness (PHEP) refers to “*the capability of the public health and healthcare systems, communities, and individuals, to prevent, protect against, quickly respond to, and recover from health emergencies, particularly those whose scale, timing, or unpredictability threatens to overwhelm routine capabilities*” [1]. The 2014–2016 West African Ebola outbreak was an important catalyst for highlighting the potential role of communities in PHEP, as well as their significant capacity to facilitate the response. It was, after all, only when the authorities put community concerns – both about Ebola itself but also about the *response* to Ebola – at the core of their thinking that control efforts during this outbreak started to bear fruit [2, 3].

For successful community-oriented PHEP it is necessary to understand how health and relevant non-health institutions collaborate with the community and what good practices exist. Such a people-centred preventive approach using engagement with relevant stakeholders is also called for in the 2015 Sendai Framework for Disaster Risk Reduction [4]. Similarly, opportunities for collaboration is also emphasized in EU Decision 1082/2013/EU (October 2013) on serious cross-border threats to health, which emphasizes the need for closely working relationships between the public sector, civil society organizations and scientific research institutions [5]. However, the practical details of how this should happen, and the specifics of who and what exactly should be targeted in community engagement activities for different health threats are still not widely recognised or practiced.

This paper reports on qualitative case studies of two tick-borne disease events, both of which took place in 2016: Crimean-Congo Haemorrhagic Fever in Spain, and Tick-Borne Encephalitis in the Netherlands. They were selected as cases for the study because they reflected the increasing incidence of emerging diseases in Europe, and thus they acted as ‘vehicles’ for examining the broader issue of community engagement in preparedness and response to such events. The study was conducted in collaboration with the national public health authorities in the two countries and with the European Centre for Disease Control (ECDC), and it aimed to identify potential or actual synergies that emerged between affected communities and relevant official institutions, prior to, during, and after the respective events. Through this, the intention has been to contribute to the development of an empirically-derived and practical approach to community engagement for infectious disease threats in the European Union.

### Community engagement

The term ‘community’ refers here to populations that may be directly affected by, or that may be at risk from the disease in question [6]. Thus, the community is seen as distinct from the government authorities who are tasked with addressing the disease. A synergistic relationship between the community and the authorities is typically captured under the label of ‘community engagement’. Community engagement has been defined as the “*direct or indirect process of involving communities in decision making and/or in the planning, design, governance and delivery of services, using methods of consultation, collaboration and/or community control*” [7]; while ‘synergies’ refer to the added value that derives from the process and outcome of two or more stakeholders or sets of stakeholders working together towards a common goal [6]. Community engagement involves more than just participation [8]. Rather, it involves a process of working collaboratively with and through groups of people affiliated by geographic proximity, special interest, or similar situations to address issues affecting the well-being of those people.

Levels of community engagement can start with a focus on a particular health issue and gradually develop into longer-term partnerships with wider aims and scope. Community engagement is a complex process because there are many stakeholder groups, each with their own interests and particular expertise. Effective community engagement identifies the relevant stakeholders in dialogue, and demonstrates a sustained respect for the values, priorities, and cultural practices of the community, while also tapping into existing community interests and priorities [9].

At present there seems to be a gap between evidence and practice in terms of such synergies and best practices in PHEP community engagement, which may adversely affect the ability of institutions and communities to be prepared for and to effectively respond to emergencies. Supportive, collaborative relationships that, from the outset, are culturally sensitive and that bring a wide range of organisations and people together will however generate a more effective response to emergencies than those that are not based on this sort of mutual respect [6].

Another factor that is recognised as being central to effective community engagement is trust. A lack of community trust in government bodies – that may be rooted in past injustices or continuing inequalities within the community [10–12], or in the institutional utilisation of scientific experts who are perceived as arrogant and out-of-touch with community priorities [13] – can constitute significant barriers to engagement. In addition, it is important to recognise that not everyone from the community gets to participate in the engagement process. Some community members may not be able to attend engagement meetings because of logistical constraints [14, 15], or they may be prevented from joining as a result of community level power dynamics [12, 16], or due to miscommunications [17].

Further, public health emergency preparedness may not be the first priority in communities, where a range of other more immediately pressing issues may need addressing before people will consider engaging in such activities [18]. Similarly, efforts aimed at community-based emergency preparedness may be seen as yet another burden for communities if not paired with an increase in funding and resources [19].

Finally, it is always important to ensure good communication between the community and any authorities that are engaged with them. This entails a coherent and consistent communication approach; genuine, two-way dialogue between community and authorities; rumour control as necessary [12]; and a comprehensive approach to disseminating emergency messages [20].

### Tick-borne case study events

The case study in Spain focused on two cases of autochthonous infection with Crimean-Congo Haemorrhagic Fever (CCHF) virus that emerged in the Autonomous Community (AC) of Castilla y León in August 2016. CCHF is one of very few tick-borne diseases that can be transmitted from human-to-human. Risk groups for CCHF include people who are exposed to ticks through their occupation or lifestyle, such as livestock farmers, shepherds, veterinarians, slaughterhouse workers, hunters, and hikers. In 2010, CCHF virus was identified in Spanish ticks during a study in Extremadura AC, a region of the country bordering Portugal. Subsequent studies did not find evidence of CCHF in ticks in Spain, but consensus existed that future sporadic human cases in Spain were possible [21].

In August 2016, a man who had been hiking in Ávila province, Castilla Y León AC, suffered an onset of high fever, abdominal pain and malaise, and was admitted to hospital. He was transferred to the intensive care unit of the Infanta Leónor Hospital in Madrid the following day, before being transferred again to the Gregorio Marañón Hospital in Madrid, where he died 9 days after first hospital admittance, with a diagnosis of liver failure brought on by hepatitis. There was no suspicion of CCHF, and consequently no measures were implemented to protect all the family members, health workers, and laboratory technicians with whom he or his biological samples had had contact. The second CCHF case was a female healthcare worker who had taken care of the first patient while in the ICU. She developed symptoms herself in the days after she had been caring for him, and went to the emergency room before being hospitalised and sent to an isolation unit. She recognised that some of her own symptoms were similar to those exhibited by the index case, and the connection between the two of them was then made. Tests were conducted, and the diagnosis of CCHF for both patients was confirmed at the end of August. The second patient made a full recovery [22].

The case study in the Netherlands focused on the first two endemic cases of Tick-Borne Encephalitis (TBE) in the country, appearing in July 2016 in the Utrecht and Twente regions. Tick-borne encephalitis, or TBE, is a human viral infectious disease involving the central nervous system. In approximately two-thirds of patients infected with the European TBE virus, symptoms are nonspecific [23], while in 20–30% of patients, a second phase of disease occurs involving the central nervous system with symptoms of meningitis, encephalitis or meningoencephalitis.

Although it has been prevalent in almost all countries of Central and Eastern Europe since 1980, TBE was previously only found in the Netherlands among people who had travelled outside the country [24]. However, a 2016 study identified TBE virus antibodies in deer as well as TBE-infected ticks in the Dutch Sallandse Heuvelrug National Park, located in the eastern region [25]. In July 2016, the first autochthonous case was diagnosed, a man who had been in the Utrechtse Heuvelrug National Parks, in the centre of the country [26]. During clinical observation, the patient gradually improved and no focal neurological deficits were present at discharge (day 37), although fatigue and mild subjective cognitive complaints remained. A second autochthonous Dutch TBE case was identified in another man in the Sallandse Heuvelrug region in mid-July 2016 [27]. By day nine the patient had improved, although tinnitus persisted. Shortly after, a suspected third case was found from the same region. However, this patient had also been in Germany during the incubation period, so it is not known whether they had been infected in Germany or the Netherlands.

Using these two events, we sought to document the perspectives and experiences of key actors with respect to synergies between affected communities and the health and relevant non-health sectors. Thereby we identify practices that could be of use for preparedness planning for tick-borne and other zoonotic health threats in the future, both in Spain and the Netherlands and other EU countries. Note that this article consolidates the key findings that have already been presented in three related ECDC technical reports [28–30].

## Methods

### Study design

We used a qualitative case study methodology [31], which, in both countries, included (a) review of published and unpublished documents; (b) semi-structured qualitative interviews with a range of experts working at national and Autonomous Community/regional level, and from both the health and non-health sectors; and (c) focus group discussions (FGDs) with representatives of affected communities. Details on the general methodology for this project can also be found in a previous publication [32].

### Documentary material

Material on the two cases was collected from two independent sources: internet databases of peer-reviewed literature, such as PubMed and Google Scholar, which were searched by our research team; and documents provided to us by each country’s ECDC National Focal Point (NFP) for Preparedness and Response, who were our key country contacts. This latter material included press cuttings collected over the course of the two events, background study reports, and links to the official websites that are concerned with tick-borne diseases. Additional documentary materials were collected during country visits to the two countries.

### Data collection instruments

A set of questions for the qualitative, semi-structured interviews and for the FGDs was derived from a literature review that had been conducted for ECDC during an earlier phase of this project [6]. The questions included issues around the availability and knowledge of protocols and guidance documents, trust between community and the authorities, communication and coordination activities, and the documentation (or not) of lessons learned. Questions were placed into the format of a theoretical preparedness cycle – with pre-incident, incident, and post-incident phases [17, 33]. Within this framework, the pre-incident phase involves preparation; the incident phase involves management, monitoring, and intervention; and the post-incident phase involves recovery and identifying lessons learned. Data collection instruments used in Spain were identical to those used in the Netherlands except that in the latter country they referred to TBE instead of CCHF.

### Interview and FGD participant categories

Categories for the interview and FGD participants were agreed upon in close collaboration with the Spanish and Dutch ECDC NFPs. The interview categories comprised technical experts in both the health and relevant non-health sectors. The FGD categories included people whose work or leisure activities involve spending time in areas where ticks may be present, such as farmers, hunters, hikers, and hikers. Because CCHF virus can be transmitted between humans, healthcare workers in Spain who had potentially been exposed to the virus prior to the two cases being diagnosed were also included.

### Study participants

Through their in-depth knowledge of the different stakeholders in their countries, the two countries’ NFPs identified relevant individuals for recruitment into the study. ECDC technical experts represented their respective, officially mandated institutions, and were thus identified based on their professional positions, while the community participants were recruited on a convenience basis, often representing an organisation to which they were affiliated. Community members interviewed were typically active in engagement with authorities and consisted of a mix of regular members and leaders of mostly small, community-based organisations. All participants were informed about the composition of the study team beforehand by mail. In order to facilitate the interview and FGD process, the questions were translated into Spanish and Dutch as appropriate and sent in advance to the participants to allow them to prepare.

A total of nine and 16 interview sessions were held in Spain and the Netherlands respectively, along with three and two FGDs, respectively. In all, we interviewed 22 people in the health sector, 12 people in non-health sectors, and 25 people participated in the FGDs – a total of 59 people. No-one refused participation or dropped out of the study. See Table 1 for a numerical summary, and Table 2 for details of the different participant categories who were included. We did not obtained demographic data on the study sample, but all participants were senior professionals and community members.

**Table 1:** Number of interviews and focus group discussions, and respective numbers of participants; by national, AC/regional, and community levels, for Spain and the Netherlands

	Health Sector Interviews (number of participants)	Non-Health Sector interviews (number of participants)	Community FGDs (number of participants)	Total number of participants
**Spain**				
National level	1 (2)	2 (4)	–	**6**
Madrid AC	1 (1)	2 (2)	1 (6)	**9**
Castilla y León AC	1 (2)	2 (2)	2 (9)	**13**
**Netherlands**				
National level	6 (10)	0 (0)	–	**10**
Regional level	3 (4)	2 (2)	–	**6**
Community level	3 (3)	2 (2)	2 (10)	**15**
**TOTAL NUMBER OF PARTICIPANTS**	**22**	**12**	**25**	**59**

**Table 2:** Interviewee and FGD participant categories in Spain and the Netherlands

	Spain	Netherlands
**National level interviews**	• Ministry of Agriculture, Fish, Food and Environment • General Directorate of Public Health • Press cabinet/Journalist	• Ministry of Health • RIVM Centre for Infectious Disease Control (CIb) • State Epidemiologists • Entomologist • Laboratories & diagnostics (RIVM)
**Autonomous Community/Regional Level interviews**	• Public Health Authority • Environmental and Animal Health • Press Cabinet or Communication to the Citizen’s Office • Human Public Health • Animal Public Health • Communication to the Citizen’s Office	• Municipal Health Services (GGD) • Forestry Service • Amsterdam Academic Medical Center • Agrarian Personnel Health Service (STIGAS) • Wageningen University and Research Central Veterinary Institute
**Focus Group Discussion with affected communities (Note: One or more people may have represented each category listed)**	• Occupational Hazards Unit • Emergency room clinician • Local Human Public Health Services • Central Human Public Health Services • Human Public Health Emergency Team • Veterinary Health Emergency Team • Hiker • Hunter • Veterinarian (focused on hunter activity) • Veterinarian (focused on livestock) • Farmers • Livestock farmer • Natural Parks worker	• General practitioner • Lyme patient organization representatives • Scouting • School representative • Private property owner • Children’s farm • Campground manager • Municipality employee • Community green maintenance worker • Local forester • City Gardner • Hunter • Herder

### Data collection and study team

Field work was conducted during intensive one-week visits to Spain and the Netherlands during November and December 2017. The agenda for the week had been organised in advance by the respective NFPs. All interviews and FGDs were conducted face-to-face, except for three interviews in the Netherlands which were held over Skype or on the phone for logistical reasons. Data collection was done at offices, in the case of professionals, or in community centres in the case of community members. The core study team consisted of two senior researchers, both male professors in anthropology fully employed at research universities, conducted the interviews or FGDs each in a different country (JK & DV), supported by a junior researcher (LC, JA) (PhD and MA levels) who took notes and asked additional questions when needed. All members of this core team were well versed in ethnographic methods and trust building. ECDC NFPs or their representatives joined all the meetings, thereby providing the opportunity for an iterative reflection and learning process over the course of the data collection period. In the first few days of data collection, ECDC representatives joined the meetings as well (ST, EW, JT) to introduce the team formally and help establish report. While the ECDC representatives and NFPs joined conversations, the process was always led by the study leads, who had a position external to ECDC.

Next to the documentary reviews, interview and focus group notes provided the core material for the analysis. Audio recordings were explicitly not made in order to motivate a safe space for participants to be open and transparent. For the same reason, notes were also not shared outside of the core study team. Thanks to the careful selection of the respondents, sufficient saturation was achieved on most of the core topics of interest, but we were also able to follow up with the NFPs on specific issues where we needed more information after field work was complete. Due to the short field visit period, data saturation was not discussed as a separate issue of concern.

### Data analysis

The documentary material provided background and context for the interview and FGD notes. Analysis of the interview and FGD notes was conducted thematically, using NVivo (for Spain) and Atlas.ti (for the Netherlands) software by the lead researcher and note taker in each country. The analysis was based on pre-defined themes within the framework of the theoretical preparedness cycle mentioned above [17, 33], with inductively emerging themes also included as appropriate. Material from the two countries was analysed independently, with an aim of identifying good practices that had been suggested by the participants as well as areas that could be improved. Final country reports were shared with the NFPs who invited participants for feedback at their discretion. The core research team (JK, DV, LC) then reviewed the two country reports together in a collaborative analysis workshop and drew out issues that were common to both countries as a means of identifying lessons that could potentially be applicable in other EU countries. These lessons were published in a separate report and again reviewed by ECDC and NFPs.

### Ethical considerations

Written informed consent was obtained from all interviewees and focus group participants, either in person or by email after a remote conversation. The expert interviewees took part purely in their professional capacity, while the FGD participants took part as representatives of a group who could be at risk of tick-borne diseases (either directly through tick bites or through hospital-based infection). No personal or demographic data were collected from any of the study participants. Participant quotations were minimized to maintain confidentiality and keep the information received anonymous.

## Results

The following key findings relate to community engagement in the CCHF and TBE events. The findings are presented without citations. This reflects the methodological choice of note taking rather than audio recording with transcripts to facilitate a safe space and prevent identification of respondents. The results are presented following the three phases identified above: pre-incident, incident, and post-incident. Note that the authorities in both countries already had in place protocols for responding to public health events, but these did not include a comprehensive set of guidelines for community engagement, and neither were they specific to the particular diseases in question.

### Pre-incident phase

#### Surveillance

Tick surveillance in both Spain and the Netherlands has, for many years, utilised community-based actors who provide the authorities with ticks, found during their various activities, for virologic analysis. In Spain, hunters are engaged in the process of collecting and sending in tick samples to the AC veterinarians who then send them on to the Ministry of Agriculture. The samples are analysed for different viruses, and the results are sent to decision makers in the Ministry of Health and in the ACs as well as to hospitals. Any virus identified in either in ticks or in animals led to intensified prevention messaging among the communities in the specific areas where it has been found. In addition to highlighting the important role of the hunters, this system provides a good illustration of the multi-sectoral, One Health approach [34] that can facilitate surveillance work with tick-borne diseases.

In the Netherlands, an innovative citizen science internet tool has been developed, the ‘Tick-radar’ (https://www.tekenradar.nl/), which provides a means for anyone who is bitten to register their location, give their contact details, and send in their ticks for analysis. Scientific results and lessons learned from this data is also fed back directly to the population of contributors through the citizen science portal. The individual who became the first autochthonous case of TBE in the country was engaged with the Tick-radar initiative, and as such, he had saved his tick which was subsequently analysed when TBE was suspected. This indicates the potential importance of such tools for epidemiological surveillance at population level, but also for early diagnosis in individual cases.

#### Established, ongoing connections between the community and the public health authorities

The authorities in both countries have direct connections with the most relevant community actors in relation to zoonotic diseases. In Spain, for example, hunters are linked to various different authorities, for example when licencing their firearms (police) and when they find and report sick animals (veterinarians). Livestock farmers are also regularly in touch with veterinarians. In both these cases, where links are already established, information and advice can quite easily be passed to (and from) at-risk groups. It is important to note, however, that not all at-risk groups may be reached through these means. Hikers, for example, are often not formally affiliated with any groups or institutions, and thus there may be no formal mechanism for dialolgue and providing them with information.

In the Netherlands, we identified an extensive network of pre-existing community-level actors and institutions engaged with tick-borne diseases, with certain key ‘brokers’ clearly identified as being more connected than others, and therefore having the potential to play a central role both in the prevention of and in the response to any future tick-borne disease event. The main brokers here include the Municipal Health Services, the RIVM Centre for Coordination and Outbreak Control (RIVM-LCI), family medicine practitioners, the Dutch Association of Lyme Patients, and the National Green Lyme Working Group (GLWG). The GLWG is a network that includes collaboration with an extensive network of “green” partners (e.g. nature owners, landscape management, wildlife, etc.), occupational health and medical providers interested in reducing tick-borne disease in their workforce, as well as a Lyme Borreliosis patient organization.

#### Urban-rural differences in levels of perceived risk

A perceived divide in the levels of concern was noted between the urban and the rural populations in the Spanish case study, whereby ticks are seen as a greater problem by those living in the city, who are at negligible risk, than by those in the rural areas, who are exposed to them on a daily basis. This applies to CCHF as well as to other tick-borne diseases. For many rural dwellers, and especially people who work outside, ticks are a regular part of life, and they have extensive experience of being bitten by them as well as good knowledge of preventive practices. For example, one of the farmers we spoke to in Castilla y Léon told of how he sometimes removes as many as 10–20 ticks after being outside for a day; while a national park worker reported that people in the area do not perceive themselves as having *problems* with ticks, even if they had tick bites. These varying levels of perceived risk could have implications for the ways in which the authorities engage with different community groups. In the Netherlands, geographically different levels of concern for TBE were not observed.

#### Hard-to-reach groups

Hikers were particularly highlighted in Spain as a heterogeneous interest group with little formal organisation and, in general, relatively limited knowledge about prevention of tick bites and tick removal. Hikers indicated that many among their peers do not wear protective gear. While some organised hikers’ groups do exist – which could theoretically be identified and targeted with information – most people go hiking into tick-infested areas without any sort of organisation to provide back-up or information.

In the Netherlands, vulnerable groups mentioned included pet owners, scouting members, schoolchildren, children in day care, garden owners, hikers, foreign tourists, and volunteers working in green areas. Many hunting, conservation, forestry or other green organizations regularly use volunteers, but without any access to occupational health services. For example, the Utrecht region Forestry Service has 230 volunteers, many of whom work in potentially tick-infested areas, but they do so without access to the occupational health services of the Service. The situation is more challenging in some children’s farms, since these are frequently staffed by temporary neighbourhood volunteers with little or no training about ticks, and who may not therefore offer good protective advice to the visiting children.

### Incident phase

#### Stakeholder analysis

No formal stakeholder analysis was conducted in Spain during the CCHF event. In the Netherlands, after confirmation of human cases, the authorities did conduct a brief TBE stakeholder analysis for risk groups, detailing the relevant relationships for people working in or otherwise using ‘green’ areas. The analysis also identified information that was needed by at-risk communities and medical care providers – such as prevention information and risk assessments respectively – as well as what these groups could offer the response team, including acting as a channel for disseminating information about risk behaviours.

#### Provision of information to the community

Information provision to the wider community during the CCHF event in Spain was based on the principles of predictability and transparency. Two inter-related strategies were used, the first being the pre-agreed use (at least in Madrid AC) of a respected scientist who would represent the AC and its positions relating to the situation in any media briefings. The second strategy entailed utilising existing and trusting relationships with individual journalists, with an aim of pre-empting any doubts in the journalists’ minds about what was happening, and thereby avoiding sensationalist reporting that could create public alarm. The overall result of these and other related efforts was media coverage that closely reflected the information provided in official press releases. People in the community were aware of CCHF, but, as intended by the authorities, they were not particularly concerned. Groups at risk of infection by ticks were not targeted with any special information, except for hospitals where staff could theoretically be infected while caring for CCHF patients. Such facilities received press releases shortly before they were publicly released so that the information could be circulated internally as appropriate.

In the Netherlands, the authorities sent out a national press release about TBE in humans 15 days after the first case was identified, and 7 days after the second case. However, media and public attention was low in both cases, including in the affected municipalities. Some non-health sector partners who had been involved in previous communication about TBE virus in deer heard about the human case through the media, instead of directly from the authorities as they reportedly would have preferred. They had been asked not to publicise the occurrence of TBE virus in deer in order to avoid anxiety among their workers, but because they had not been officially informed about the human TBE cases, they reported that they had felt inadequately prepared for any potential dialogue with concerned members of the public.

#### Community perceptions of the incident

We were told in Spain that insufficient human resources hindered efforts by the authorities to monitor social media and thereby ascertain if there were rumours or misinformation circulating in the community about the CCHF event. The communications team at the Ministry of Health does receive alerts from a range of official sources, and they are able to focus these by using hashtags relating to particular topics. Rumours may also be reported informally through contacts with journalists who are seeking clarification or comment on an issue. However, this is not a systematic process, and while no significant rumours were reported on social media regarding the CCHF event, it is nonetheless possible that rumours were in fact circulating but these were simply not picked up.

In the Netherlands, by contrast, resources are available for RIVM to run a social media rumour management system, using software and a dedicated communications team. No significant misperceptions or rumours were reported during or since the TBE event.

### Post-incident phase

No overall systematic evaluation or After Action Review was conducted either after the CCHF event in Spain, or after the TBE event in the Netherlands, although specific lessons learned have been identified and acted upon in both countries. In Spain, the absence of a comprehensive review was reportedly because of limited financial and human resources, but informal evaluations of particular issues were nonetheless conducted at both national and AC level. These led to changes in laboratory protocols, CCHF virus surveillance systems, and the provision of public information about tick removal. In the Netherlands, the emergence of TBE remained under research at the time of our field work, and the event was officially considered to be still ongoing. Nonetheless, the authorities have worked to increase awareness among medical stakeholders in order to facilitate early diagnosis of any new TBE cases, and they have also adapted public education about tick-bites so as to include more TBE-specific information.

## Discussion

This section presents a set of good practices for promoting effective community engagement in the area of public health emergency preparedness in relation to tick-borne diseases and/or other emerging zoonotic diseases. While several of these points have been individually identified in previous work [35–37], much of the published material on this topic is from global level, the USA, Australia, or the West African Ebola outbreak, with relatively little specifically from Europe. Further, the good practices presented here are empirically derived from the data collected during this study, and they emerged through the discussions with our respondents as points that could be *collectively* considered in preparedness and response to zoonotic diseases.

Much of what is presented below is associated with activities in the pre-incident phase, but some of it may also be applicable during or after an incident. Whichever phase a given activity is connected to, it would be important to subject it to formal evaluation wherever it is implemented in order to expand the evidence base on community engagement activities within a European setting.

### View the community as a partner for optimising preparedness planning and response actions

An over-riding principle that has emerged from this study is that at-risk communities understand their challenges better than anyone else and are best place to propose effective preventive practices. Ongoing dialogue with community representatives is crucial to identify needed areas of improvement in surveillance, preparedness, and response.

### Conduct a stakeholder analysis of all community-based actors who may be involved in or affected by a zoonotic outbreak or event

A stakeholder analysis of community-based actors who may be involved in or affected by a zoonotic outbreak or event can be constructed by asking all known stakeholders to map out their connections, and then snowballing from there until no new stakeholders are identified. The analysis should ideally include details about who conducts which activities, with whom, and how, as well as which stakeholders act as brokers between various components of the network. In addition, it may be useful to map out what type of information key stakeholders (e.g. brokers) would need from the authorities, as well as what resources they can offer the authorities in the case of an emerging outbreak [38].

### Utilise pre-existing networks within the community in order to spread information about who may be at risk of tick-borne or other zoonotic diseases

The systematically-conducted stakeholder analysis described above may be the best way for the authorities to identify and then work with pre-existing networks in the community. However, the authorities will often already know and have contact with many of the main actors, and thus they may have the basis for providing information to people at risk of infection. Such networks can be utilised to facilitate the effective provision of prevention information to people who are potentially at risk of infection. However, it is important that any disease-specific community actors (such as those concerned with other, non-CCHF/TBE tick-borne diseases) are informed about the different risk profiles of these closely related diseases, including with respect to any differences in transmission risk and disease virulence.

### Cultivate and maintain relationships between zoonosis researchers and community-based monitoring networks such as hunters and foresters

Relationships between zoonosis researchers and community-based monitoring networks have proven critically important in the tick surveillance systems of both Spain and the Netherlands. However, continuous participation of community-based actors cannot be taken for granted in this work. Continuous and regular feedback should be provided to people who provide the ticks and other relevant information to the authorities, as people may be more collaborative with the authorities if they receive regular updates on the usage of the datasets to which they are contributing. This could take the form of, for example, sending them annual summaries showing the geographical patterns of tick infestation in their area or in the country as a whole.

### Identify and engage with hard-to-reach but potentially at-risk groups

There are several identifiable groups who are potentially at risk from tick-borne diseases but who, for a variety of reasons, may be hard to reach with information about prevention and treatment-seeking behaviour. These groups include hikers, foreign tourists, pet owners, scouts, schools, day care centres, garden owners, and volunteers working in nature reserves and other similar areas. Other groups could be identified through a systematic stakeholder mapping exercise, as described above.

Risk communication campaigns need take into account the needs and language abilities of target groups. For example, the suggestion was made to reach out to hikers through park information signs at parking lots, including website links to public health authorities using for example scannable QR.

### Ensure transparency in communications with the community about ongoing processes

Communication with the community and/or specific at-risk groups should be, and should be *seen* to be transparent, coherent, and consistent. It is well recognised that consistent and regular use of a trusted, media-trained spokesperson, who may become the ‘public face’ of the official response, is an essential component of efforts to manage the community response to a zoonotic disease incident or outbreak [39]. Part of the communication activities could include, for example, updates on research, even when results are not yet conclusive.

### Ensure that systematic efforts are made to monitor community perceptions of any public health incident, including through social media

By monitoring community perceptions of an issue, it may be possible for the authorities to respond to any misinformation or rumours that emerge. This can be accomplished by following specific hashtags on social media, or by noting the questions asked and the points raised to telephone hotlines, and then providing responses to these under Frequently Asked Questions (FAQs). Such a process can also help to identify new cases or clusters, to which the authorities can then respond [40].

### Recognise the rural-urban divide in perceptions about tick-borne diseases

Efforts should be made to establish an understanding of the perceptions of different categories of people – for example, urban and rural populations. Wherever differing perceptions are found, the different populations should be targeted with adapted messages, and possibly disseminated via different channels. As in all risk communication, one size will not necessarily fit all, and audience segmentation may be necessary [41].

### Conduct post-incident evaluations so that lessons learned concerning efforts aimed at promoting community engagement can be integrated into SOPs and protocols

Efforts in all relevant sectors to conduct formal evaluations after a public health incident can ensure that institutional memories are sustained and that lessons learned are formally documented and thereby remembered over the longer term [42, 43].

#### Study strengths and limitations

A wide range of professional backgrounds was represented by the interviewees and focus group participants in both participating countries, and stakeholders were also included from each of the national, AC/provincial, and community levels. Further, the semi-structured qualitative interview and FGD methodology that we adopted in this study permitted participants to discuss issues as *they* wanted to, and to raise topics that *they* felt were important. We are therefore confident that while we may not have achieved saturation on every relevant theme, the major issues were all addressed.

Data collection took place in both countries just over 1 year after the respective public health events, so not all the details may have been recalled accurately by the respondents. However, these were significant events for all the respondents, and we found that people had much to say about them and their own engagement. Given the range of respondents interviewed and the detail with which they spoke, we are confident that the issues presented above are broadly reflective of all the core issues on the topic.

In both countries, the research team was accompanied to the interviews and FGDs by representatives of the national public health authorities. While the presence of these staff could perhaps have affected the responses of some interviewees or focus group participants, we consider it unlikely that it brought about any significant biases in our database. Most issues were discussed with several respondents, and while consensus was not achieved about everything, the study participants generally complemented each other’s content rather than contradicting it. Further, the learning obtained from the involvement of national level representatives during local level discussions may have strengthened collaboration and understanding between the levels.

## Conclusions

The two events studies in this paper were, in many respects, very similar in occurrence. Both dealt with a tick-borne, zoonotic disease in which many stakeholders were involved; both had similar timelines and periods of occurrence; both received low media and public attention; and both came as relatively surprising events for the authorities involved. Our findings emphasize the importance of trust between the affected communities and institutional authorities, facilitating community ownership of (aspects of) the preparedness and response activities, targeting and supporting vulnerable populations, ensuring consistent and transparent communication, and using existing networks. The clearest example of the latter is in community based surveillance, in which hunters, farmers and foresters actively cooperate with authorities to detect threats which are of key relevance to their own occupational health. Another examples are the citizen science projects, illustrated by the Dutch Tick-Radar project, which allow citizens to make a real contribution to prevalence studies, and the quick engagement of civic tick-born interest groups, such as the Green Lyme Working Group in the Netherlands, in the response to a new tick-born disease.

While external expertise may be needed to coordinate these activities within the community, ownership of them is not exclusively located with the authorities. Facilitating public health emergency preparedness synergies requires an awareness of the specific role each partner may have. It also entails recognition of the fact that different types of expertise reside at all levels. While for some this may be clinical, epidemiological, or environmental biology, for others it may be about the volunteers at risk in a forest, or the way in which children interact with nature during school outings. All of these experts can contribute when an emergency response is orchestrated, not alone, but together. This principle would apply to any sort of public health emergency, whether at the pre-incident, incident, or post-incident phase of the preparedness cycle.

## Data Availability

The interviews used and analysed for the current study were conducted with a range of government and AC/provincial level officials from the two participating countries, and as such the datasets cannot be made publicly available. However, the datasets are available from the corresponding author on reasonable request, and after consultation with the ECDC National Focal Points for Preparedness and Response for the two participating countries.
